# Cavitation detection for brain imaging and therapy

**DOI:** 10.1186/2050-5736-3-S1-O38

**Published:** 2015-06-30

**Authors:** Meaghan O’Reilly, Ryan Jones, Kullervo Hynynen

**Affiliations:** 1 Sunnybrook Research Institute, Toronto, Ontario, Canada

## Background/introduction

Cavitation-mediated therapies for the brain, such as ultrasound-induced Blood-Brain barrier opening and sonothrombolysis for the treatment of stroke, are being increasingly investigated. Robust methods for monitoring and controlling cavitation are necessary for safe translation of these techniques into clinical practice. This talk will review our work detecting and mapping cavitation activity in the brain, as well as using the cavitation signals to control treatments. The potential application of these techniques for mapping the vasculature will also be discussed.

